# Development of information dissemination methods that contribute to improving maternal and child healthcare using social networking sites: a community-based cross-sectional study in Japan

**DOI:** 10.1186/s12889-022-12877-8

**Published:** 2022-03-11

**Authors:** Sayaka Ikeda, Yutaka Ueda, Asami Yagi, Mariko Taniguchi, Satoko Matsuzaki, Tsuyoshi Takiuchi, Ai Miyoshi, Hitomi Arahori, Kei Hirai, Tadashi Kimura

**Affiliations:** 1 Department of Social and environmental medicine, Division of Environmental Medicine and Population Sciences, Osaka University Graduate School of Medicine, Suita, Osaka, Japan; 2 Department of Obstetrics and Gynecology, Osaka University Graduate School of Medicine, 2-2, Yamadaoka, 565-0871 Suita, Osaka, Japan; 3 Department of Pediatrics, Osaka University Graduate School of Medicine, Suita, Osaka, Japan; 4 Department of Clinical Psychology, Osaka University Graduate School of Human Sciences, Suita, Osaka, Japan

**Keywords:** Mothers, Infants, Raising children, Loneliness, Social networking site (SNS), Social support

## Abstract

**Background:**

In recent years, feelings of isolation among mothers caring for small children has become a significant social issue in Japan. The purpose of this study is to develop a message to alleviate their loneliness, to evaluate the impact of social networking sites (SNS) for delivering such messages, and to propose means of more effective information transmission to promote health for mothers raising small children.

**Methods:**

Our study was conducted in two stages, first an interview and then a cross-sectional study of the mothers involving a questionnaire survey. The interview was targeted two public-health nurses caring for mothers. Based on these interviews, we developed six messages intended to alleviate the mothers’ sense of loneliness, which were vetted by seven mothers. The second stage was to conduct a questionnaire survey of mothers both before and after our selected message as advertisement on Instagram and analyzed the effect. The surveys were collected during routine child health check-ups in the City of Takatsuki, Japan.

**Results:**

From the six draft messages created based on interviews with public health nurses, we selected the message that most relieves the feeling of loneliness of the mothers who are raising small children.

The survey questionnaire was taken by 494 mothers prior to our posting of Instagram advertisements (ads), and afterwards by 419 mothers. The percentage of mothers feeling loneliness tended to decrease after reading the messages (before ads.:8.1%, after ads.:5.8%). 8.6% of the mothers (36/419) remembered seeing the Instagram ads. Mothers with financial anxiety were significantly more likely to have remembered seeing the Instagram ads (*p* < 0.01).

**Conclusions:**

Our results indicate that usefulness of SNS messaging for mothers raising small children may reduce their feeling of loneliness. Among the SNS, disseminating child-rearing information on Instagram may be more effective for people with financial instability.

**Supplementary Information:**

The online version contains supplementary material available at 10.1186/s12889-022-12877-8.

## Background

In the past in Japan, young mothers were strongly supported by both their families and their communities in the upbringing of their young children [1] However, in recent years, the feelings of isolation of mothers bearing infants has become a significant mental health issue in Japan. It has been pointed out that women constitute a greater risk group for loneliness than men [2]. Especially, mothers who stay at home with their small children are recognized as a high-risk group for loneliness [3], it has been reported that the risk of loneliness among mothers with infants is particularly higher in Japan compared to other OECD countries [4]. These feelings of loneliness have many contributors. The trend toward nuclear family, the financial insecurity, the father’s non-participation in the child-rearing process because of longer job and mothers have to bear more responsibility and childcare burden in mentally and physically [4, 5]. The dilution of neighborhood relationships because of the transient and impersonal nature of modern communities is also a large part of the problem [6–8].

Loneliness is best defined as the perception of an unpleasant experience that occurs when a significant quantitative or qualitative deficiency occurs in a network of personal social relationships [9]. The feeling of loneliness felt by a new mother is an important social issue that can have a significant impact not only on her own health but also on the health of her child, and on society as a whole. There are numerous studies that have focused on maternal- and postpartum- depression [10, 11]. However, the study of the delayed loneliness of mothers with small children has been limited.

Many studies have suggested that the social network, social support, and social environment have larger effects on the health of women than of men [12]. Social capital is a concept related to social support. Simply explained, social capital is the benefit, both social and personal, that is experienced by having social relationships with others [13]. In pregnant and postpartum women, higher individual-level social capital is associated with lower self-reported depression symptoms [10].

Social networking sites (SNS) are defined as “web-based services that allow individuals to (1) build a public or semi-public profile within a bounded system, (2) articulate a list of other users with whom they wish to share connections, and (3) view and traverse the list of connections in the system” [14]. Facebook, launched in 2004 and one of the most widely used SNS, is currently reported to have 2.8 billion global users, with 26 million users in Japan. [15]. Instagram and Twitter are gaining in popularity, with 1.22 billion and 350 million global users, and 33 million and 45 million users reported in Japan, respectively [16, 17]. In particular, the number of users of Instagram is increasing rapidly, and the usage rate of women in their 20s to 40s, who are of the child-rearing generation, is high in Japan [17]. In fact, mothers, in particular adolescent mothers, are increasingly using SNS to collect child-rearing information [18]. However, there have been only a limited number of reports on the usefulness of SNS in the area of new maternal and child health.

The feelings of loneliness of mothers with infants varies greatly, depending on the age of the mother and the child. For example, the first-time mother of a 4-month-old child is usually not accustomed to a life with infants, with repeated breastfeeding, diaper-changing, and sleep deprivation, and are thus prone to anxiety and depression. The purpose of this study was to develop messages to help alleviate an infant’s mother’s sense of loneliness and to evaluate the impact of using SNS advertisements to improve the effectiveness of information transmission that promotes their health and wellbeing.

## Methods

In order to evaluate the effectiveness of providing health information using SNS to improve the health of mothers raising 4-month-old children, this study was conducted in two stages: an interview of public health nurses, mothers and a questionnaire survey of mothers which is intervention study. This study was conducted with the mothers raising 4-month-old children scheduled to have a routine health check-up in Takatsuki city, a northern suburb of Osaka, Japan. Takatsuki City has a population of roughly 351,000, with an annual birth rate of about 2,500.

### 1st Stage: interview

In-person interviews were first conducted with two public health nurses. We used their input to produce six messages designed to reduce the anxiety of the mothers of infants, then we surveyed seven mothers to evaluate the effectiveness of the various messages. Using that evaluation information, we developed an ad to run on Instagram.

### For public health nurses

In order to develop a message to alleviate their feelings of loneliness and anxiety of mothers with infants, we sought to gain an objective understanding of the mother’s perceived worries. To get that objectivity, we first interviewed two public health nurses, one in Yao City and the other in Takatsuki City, who were in charge of maternal and child health and who were routinely consulted by the mothers we were studying. We interviewed the nurses for 60 min apiece. We asked them about the following:

・What are the common concerns of the pregnant women and mothers of newborns that you see? Not limited to their consultation with local government programs, but generally: What kinds of problems are they having, who they are consulting with, what were the characteristics of the mothers who can, and who cannot, consult with someone, etc.?

・Regarding the system for accepting consultations with local government programs, what is the timing for when there are many consultations from the mothers, what are the main contents of these consultations and what are their responses, what are the characteristics of the mothers who do consult with local governments, and how do they perceive the role of local government programs, etc.?

・What are the things public health nurses want to educate pregnant women and mothers of newborns about?

### For mothers raising 4-month-old children

**Fig. 1:**
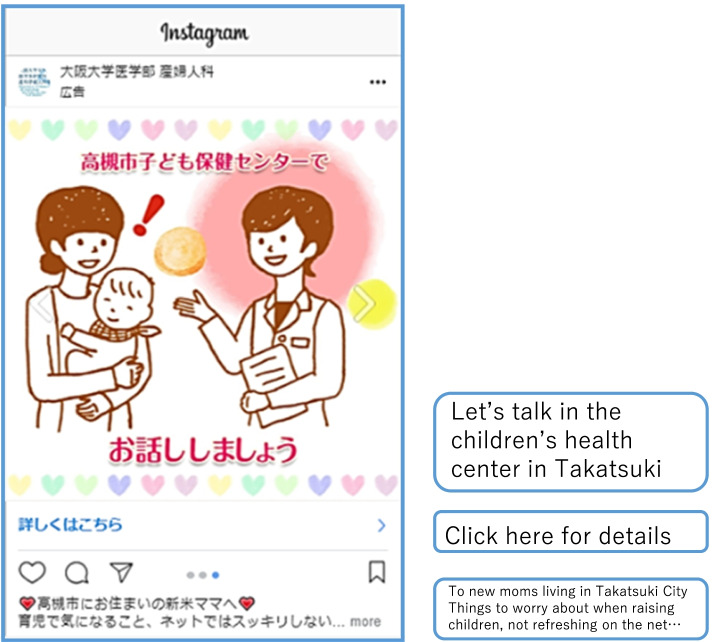
Instagram ads designed to alleviate an infant’s mothers’ feelings of loneliness

Based on our interviews with the public health nurses, we developed six types of messages for mothers with infants meant to reduce their anxiety and worries. Each message was presented to the seven mothers who represented the intervention targets for this study (Table 1). In addition to exploring how they felt toward to the six types of messages and how it improved their attitudes, we also obtained personal information on their own anxieties and worries about child-rearing, when those negative feelings arose, and how those apprehensions were resolved. Interviews with the seven mothers were conducted on July 16th and 17th of 2019. Based on these seven interviews, we developed an Instagram ads designed to alleviate an infant’s mothers’ feelings of loneliness (Fig. 1).

**Table 1:**
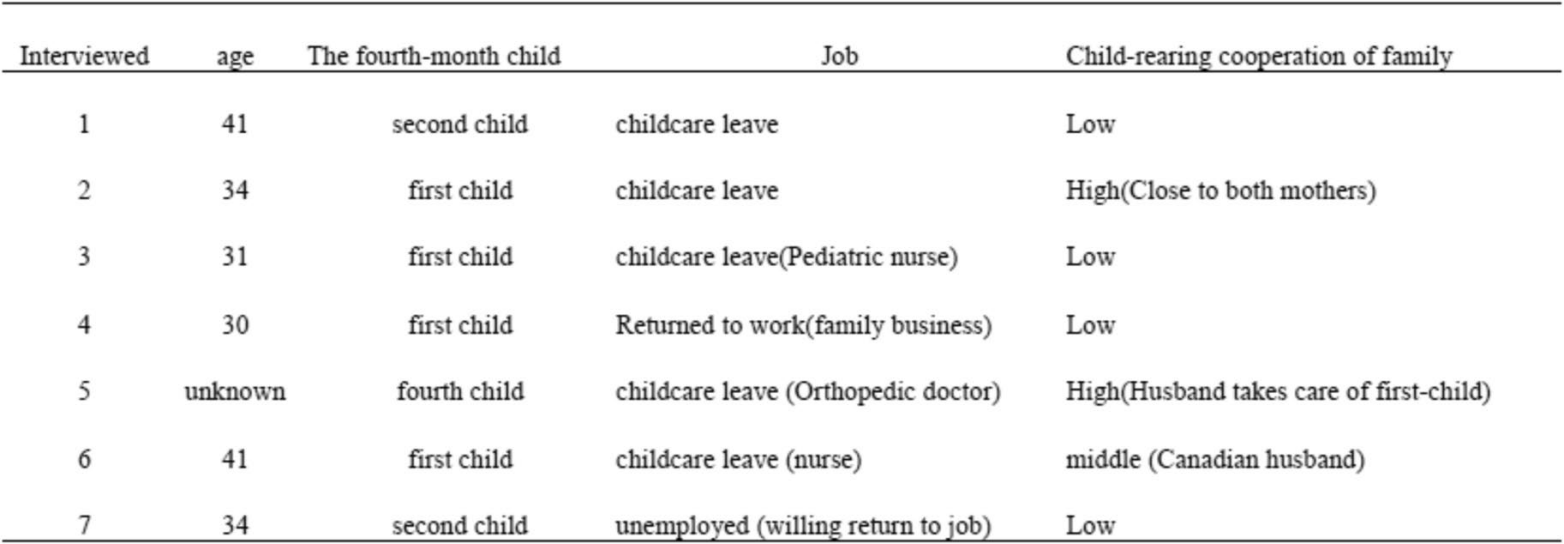
Information obtained from the seven interviewed mothers with fourth-month child

### 2nd stage: questionnaire survey

#### Study participants

This part of the study used the opportunity provided by the routine health check-ups for 4-month-old children in Takatsuki City to conduct a survey of their mothers about their concerns, and after posting of Instagram ads, to evaluate the impact of the ads to evaluate the impact of using SNS ads to improve the effectiveness of information transmission that promotes their health and wellbeing. We recruited 565 mothers who would participate in a 4-month child health check-up from June to August 2019, and 632 mothers from January to March 2020 the following year, and they were all eligible. From June to August 2019, 505 mothers answered the questionnaire, and from January to March 2020, 428 mothers answered the questionnaire, and the questionnaire collection rate was 89.4% and 67.7%, respectively. We deleted 11 surveys before Instagram ads and 9 surveys after Instagram ads those who did not answer correctly. As a result, the number of the questionnaire analyzed in this study was 494 mothers before Instagram ads and 419 mothers after Instagram ads.

Instagram ads were posted between the questionnaire from December 2019 to January 2020. The Instagram ads were sent to all mothers aged 18 to 45 in Takatsuki City. The ads included guidance for contacting local parenting support desks and a message to help alleviate their sense of loneliness and helplessness. At one month prior to their check-up, a survey guidance sheet and a questionnaire were send by ground mail to the home of the targeted mothers. Survey participation was voluntary and anonymous, so those who completed the questionnaire were considered to have been informed and knowingly consented to participate in this study.

### The content of questionnaire

The questionnaire was developed based on the results of our baseline interviews with nurses and infant mothers and also on previous studies on loneliness. The questionnaire contained 17 items, with the following content: The basic characteristics of the respondent, childcare status of the children, types of communication devices and social networking sites used, feelings of self-efficacy about her own health and their child’s health (Supplementary 1). We used an anonymous self-reporting questionnaire, which was collected directly from the mother at the reception counter of the public health center where the health check-up was conducted.

### Statistical analysis

This is a community-based cross-sectional study. The chi-square test was used to compare background factors. We compared predictors that promoted a reduction of the sense of loneliness among mothers before and after receiving our Instagram ads. In the questionnaire given to the post-Instagram ad group, we analyzed the differences between those who remembered seeing the advertisement and those who did not. The responses to the questions about confidence, loneliness, and efficacy were sorted as follows: If the answer was either “often” or “sometimes”, we regarded the answer to be Yes. If the answer was “Neither”, “Don’t feel much” or “Not at all”, we regarded it to be No. In addition, we asked the mothers about the type of social networking sites they were using at the time. We compared each item between the two groups and the p-values were calculated using a chi-square test. The level considered to be statistically significant was set at *p* = 0.05. All statistical analyses were carried out using STATA version 14.0 SE software (Stata Corp LP).

## Results

### 1st stage: interview

#### Interview of two public health nurses

We found that almost all pregnant women had some contact with local government public health nurses in charge of maternal and child healthcare. This contact was triggered by the issuance of maternal and child healthcare handbooks and continued through newborn home visits, postnatal examinations, and infant health check-ups. In both Yao City and Takatsuki City, a very high percentage (40–50%) of the new mothers were monitored by public health nurses.

There were a wide variety of worries expressed by the mothers to the nurses during their check-up visit, but the most frequently stated concerns were about breast milk, the baby crying or not sleeping at night, mastitis, the mother’s inability to sleep, and whether the infant was growing properly (weight and the stage of holding their head up), when the physical condition of the child would require going to the hospital, vaccinations, and baby food (not eating or the exact amount that should be eaten). The characteristics of the mothers that the public health nurses were particularly worried about were those who had had a long and/or prominent career. It was also found that people with low self-affirmation tended to have elevated levels of anxiety, and that those with strong beliefs were often worried.

**Fig. 2:**
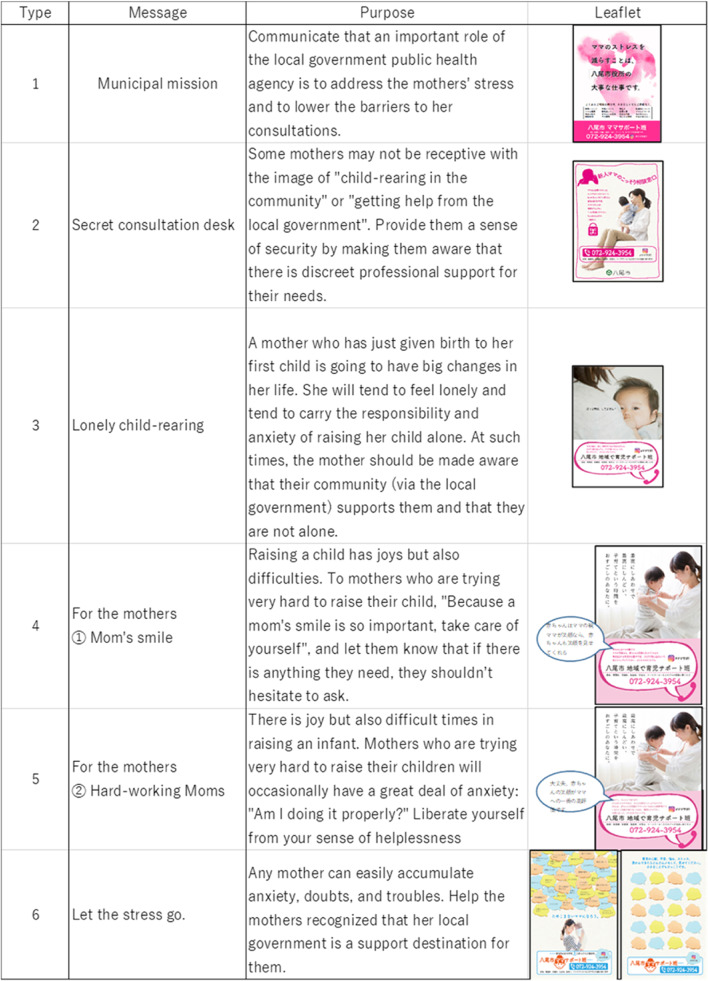
Six types of message leaflets intended to reduce an infant’s mothers’ anxiety

Based on the public health nurse interviews, we developed six types of message leaflets intended to reduce an infant’s mothers’ anxiety (Fig. 2):

- Type 1 Municipal mission: Communicate that an important role of the local government public health agency is to address the mothers’ stress and to lower the barriers to her consultations.
- Type 2 Secret consultation desk: Some mothers may not be receptive with the image of “child-rearing in the community” or “getting help from the local government”. Provide them a sense of security by making them aware that there is discreet professional support for their needs.
- Type 3 Lonely child-rearing: A mother who has just given birth to her first child is going to have big changes in her life. She will tend to feel lonely and tend to carry the responsibility and anxiety of raising her child alone. At such times, the mother should be made aware that their community (via the local government) supports them and that they are not alone.
- Type 4 For the mothers ① Mom’s smile: Raising a child has joys but also difficulties. To mothers who are trying very hard to raise their child, “Because a mom’s smile is so important, take care of yourself”, and let them know that if there is anything they need, they shouldn’t hesitate to ask.
- Type 5 For the mothers ② Hard-working Moms: There is joy but also difficult times in raising an infant. Mothers who are trying very hard to raise their children will occasionally have a great deal of anxiety: “Am I doing it properly?“ Liberate yourself from your sense of helplessness.
- Type 6 Let the stress go: Any mother can easily accumulate anxiety, doubts, and troubles. Help the mothers recognized that her local government is a support destination for them.

#### Interview with mothers raising 4-month-old children

##### Base-line interview with seven mothers

The seven mothers interviewed in this study, even if they answered “Nothing in particular” to the question “Have you had any problems or anxiety about childcare?“, still told us that there was at least one time when they felt anxiety, loneliness, and became unstable. Their comments related to each message leaflet were as follows:

- Type 1 message - Municipal mission: This leaflet was not very interesting to them because it left the impression that the local government wouldn’t actually do anything, and also the agency mission had nothing to do with the interviewed mother.
- For the Type 2 message - Secret consultation desk: The interviewees felt that it was true that searching for a solution on the Internet didn’t always find the correct answer. My friend saying “I have never bothered doing that” made them feel that “I care too much”. They were grateful that they could talk about even little things without worrying about it becoming public. The leaflet gave them the impression of the hurdle to reaching out to the consultation desk was being lowered.
- For the Type 3 message - Child-rearing can be lonely: In the interview, it was said that the message evoked the image of a mother who is in poor mental condition would be the one seeking consulting. On the other hand, there was also an opinion that there was a perception that doctors could provide mental health care, but they said that the hurdle for a doctor consultation seemed to be raised.
- Toward the Type 4 message - “For the mothers: ①“Mom’s smile”, there was an opinion that because the idea “Baby is a mirror of mom, so if mom smiles, the baby will smile too” was too correct because it would also mean the painful reality that the baby would suffer if the mother’s mental condition became poor.
- The Type 5 message - “For the mothers: ②“Working hard moms” The more moms do their best, the more they worry about “Am I doing it properly?“ The message was considered to be true, and some moms were encouraged by it.
- About the Type 6 message – “Let go of the stress!”: Regardless of whether or not we mothers actually call, there was a voice saying that we can feel relieved to know that there is a place that will listen to the our stories. On the other hand, there was one comment: “I don’t feel like discussing specialized matters with a “consulting desk that listens to complaints.“ There was another negative opinion, “Why would you consult with a local government office?“ It was clear that the fact that knowledgeable professionals at the help desk would be providing appropriate and accurate information and advice was not being properly conveyed by the message.

Based on these comments, we created Instagram ads that were intended to alleviate a mothers’ sense of loneliness.

### Questionnaire

#### Characteristics of the study participants

The questionnaire was used to survey 494 mothers before the Instagram ads were posted and a 419 mothers after the posting of the ads. The surveys were handed in at Takatsuki City health clinics when the mother and infant attended their routine 4-month health check-ups. Table 2 summarizes the basic characteristics of all participants. The mean age was 32.9 years for both survey groups. There were no significant differences between the before and after Instagram ads survey groups with respect to the sibling position of the child having the health check-up, the family’s financial insecurity, or in having any mental health problems.

**Table 2:**
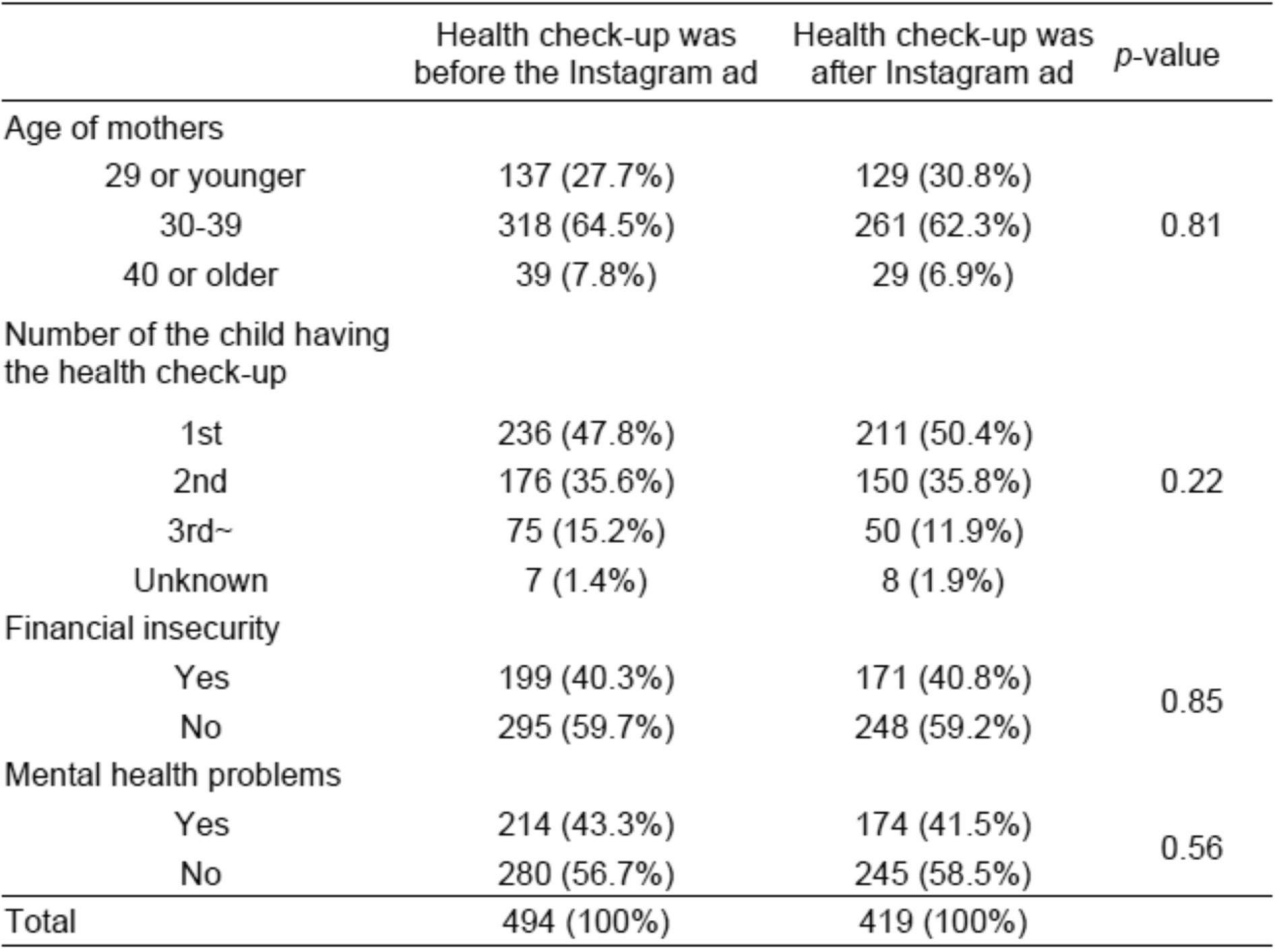
Participant characteristics

### The impact of the Instagram ads

A comparison of the questionnaire results before and after the sending of the Instagram ads is shown in Table 3. Their awareness of local child-rearing support desks and their feelings of self-efficacy in their child-rearing were not significantly different between the two groups. However, there was a tendency for the percentage of mothers feeling loneliness to decrease after receiving the Instagram ads, although the difference was also not statistically significant.

**Table 3:**
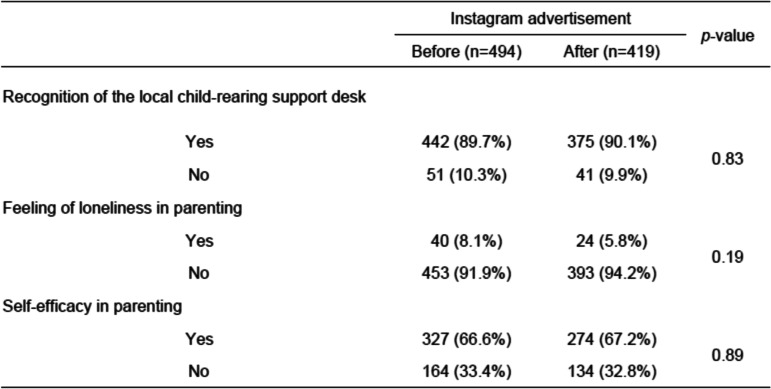
Comparison of questionnaire results from before and after sending Instagram ads

The two survey groups’ awareness of the Instagram ads are shown in Table 4. Of the 419 mothers in the post-Instagram group, only 20 (4.8%) remembered seeing the ads. In addition, 16 women (3.8%) had heard of the ads, although they had not seen them. Thus, totally 36 of the mothers (8.6%) recognized the Instagram ads in some way. Table 5 shows the characteristics of the mothers who remembered seeing the Instagram ads. Survey participants with financial anxiety were significantly more likely to remember having seen the Instagram ads (*p* < 0.01). There were no significant differences in the responses surrounding mental health problems, self-confidence, feelings of anxiety, dark feelings of failure, and not feeling that things were working as planned concerning health.

**Table 4:**
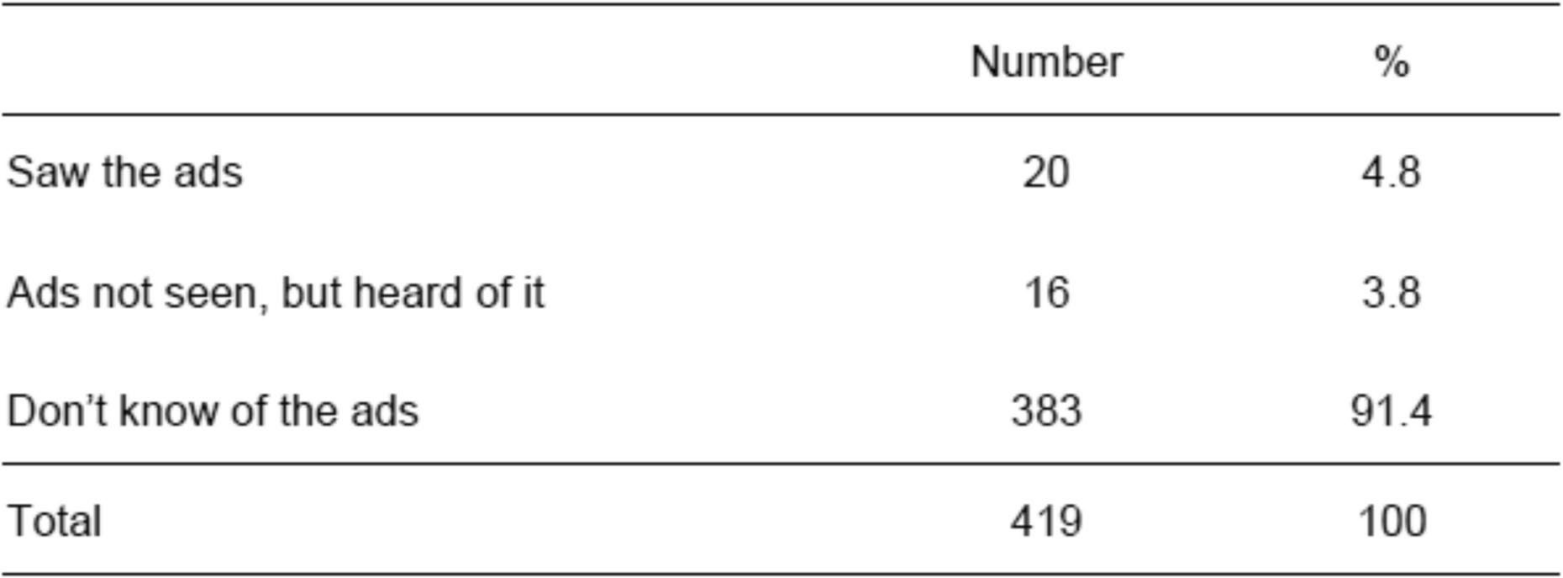
Awareness of the Instagram ads

**Table 5:**
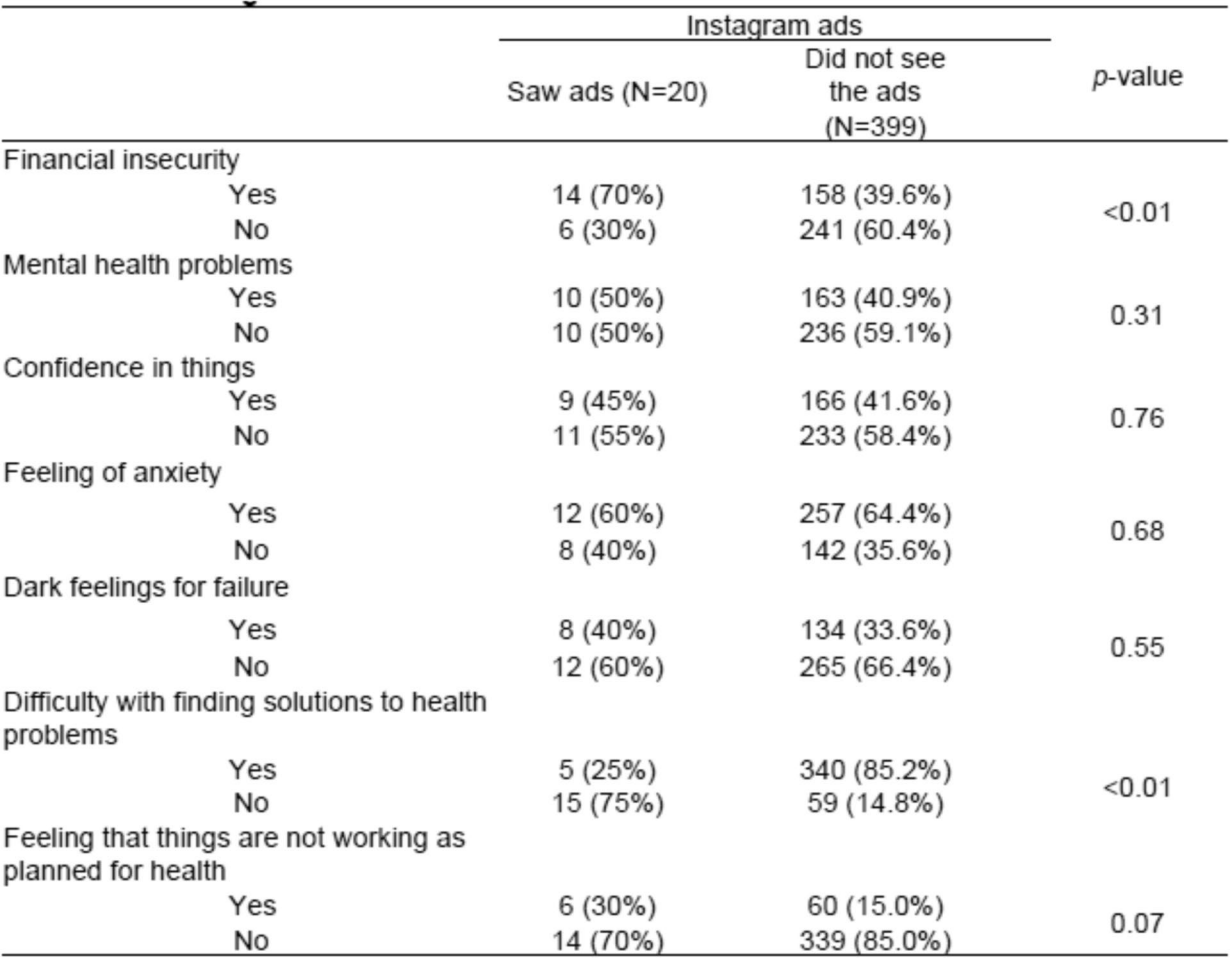
Characteristics of mothers who saw the Instagram ads

### The types of SNS device used by the mothers

Table 6 shows the results concerning Instagram ad recognition and types of SNS being used. Significantly more of the 20 women who saw the Instagram ads used Instagram on a daily basis (*p* < 0.001). We found that 70% of these women also used Twitter (*p* < 0.001) and 50% (*p* = 0.23) also used Facebook. Most importantly, we found that 90% of the 20 participants who saw the Instagram ads, and 91% of 399 participants who didn’t see Instagram ads, used LINE on a daily basis.

**Table 6:**
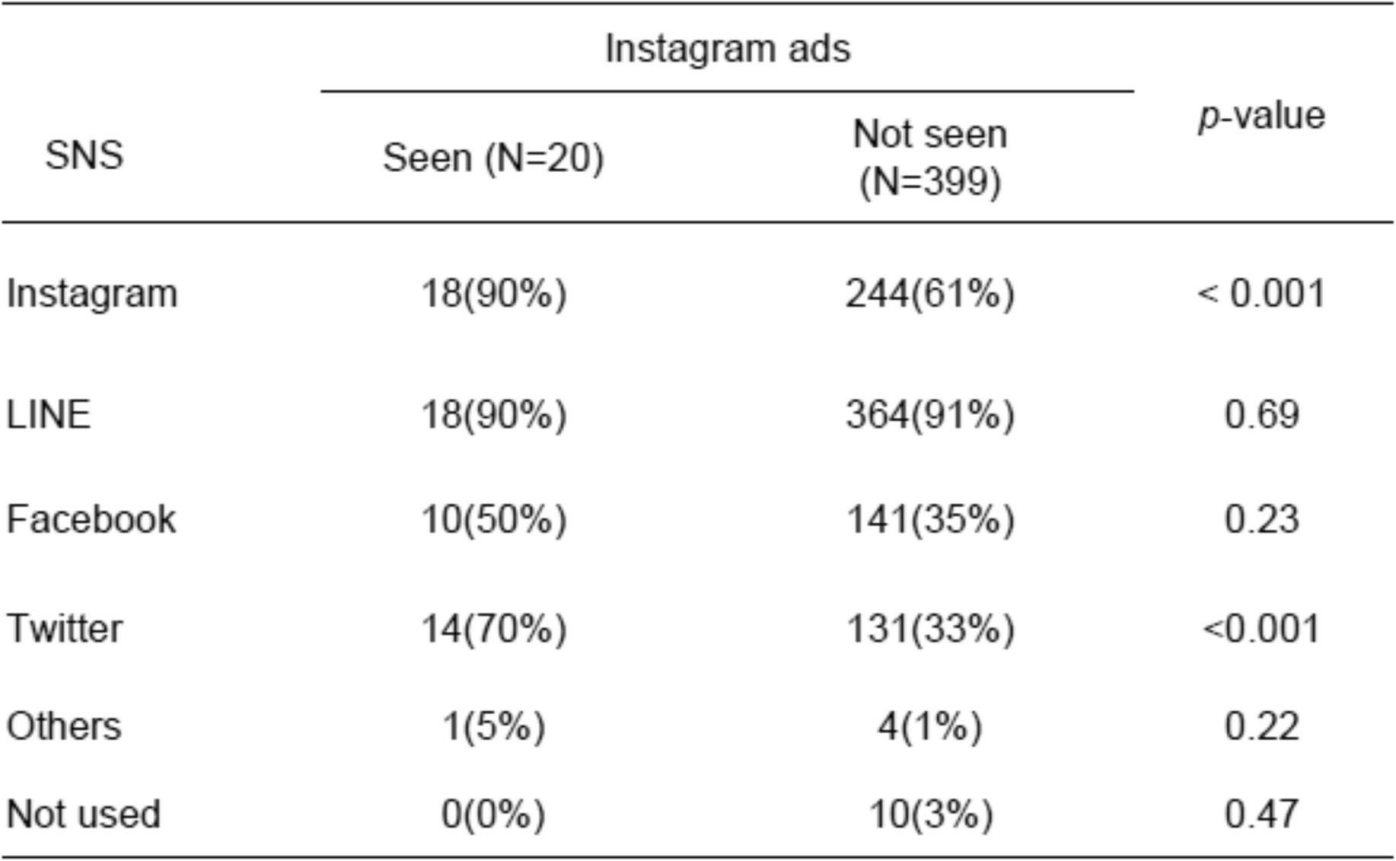
Instagram ad recognition and SNS types

## Discussion

In present study, we first conducted two types of baseline interviews, one with front-line pubilc health nurses and one with seven mothers of the targeted audience, and created an effective message to alleviate the feeling of loneliness of the infant’s mother. Next, since there was no research that evaluated the effectiveness of using SNS to disseminate health information from local governments, we conducted a questionnaire survey of the subjects of the health check up before and after posting the created Instgram message. By assessing the impact of messages delivered via SNS, we considered effective methods of communicating information to promote the mental health of mothers.

First of all, in the interview with the public health nurses, the nurses particularly worried about mothers who was relatively older, or who had a long and prominent career prior to the arrival of the infant. Studies by Mathiesen [19] and Sperich et al. [20] showed similar results, indicating there was often increased psychosocial stress associated with higher-educated mothers. This is because mothers with a higher education and carrier found it more stressful to balance both their job and family demands. Such women are used to making judgments and responses for themselves, so they are normally able to control their situation. However, while trying to do ‘everything perfectly’, they were also not very good at relying on other people. It was also found that mothers with low self-affirmation tended to have elevated anxiety, and those with delusions were also often worried. As an important issue for future consideration, we found that recognition that the local government is now a place supporting child-rearing is not widespread, i.e., it has not yet become “a place where you can easily rely on when you are in trouble raising your children.” The local government needs to do a better of conveying their message: “Child-rearing is not something that you do alone; you, your family, and your community will do it together”. The importance of the work of health professional has been mentioned in previous studies [21], and it is thought that communicating the role of the local government in supporting child rearing might help reduce the social and emotional loneliness of mothers.

Next, from the interview with the seven mothers having a 4-month-old child, the following four things should be points of emphasis to convey in messages to other mothers: To improve their self-affirmation, we should tell mothers who tend to feel anxious that they are doing OK; we should provide the new mothers with the comforting perception that they can consult with local help desks that can provide them with correct and appropriate information; this will help them see the merits of consulting with their local government health offices, and it will lower the perceived hurdle to overcome for seeking consultations with their local government health office. The general definition of social isolation consists of a lack of social networks or the feeling that they are not part of a social group. In turn, emotional loneliness refers to the lack of intimate attachment to others [22]. Therefore, it is thought that by communicating a message to mother with a small child and consulting to a local help desk with building a good relationship, it will reduce social and emotional loneliness.

From the questionnaire survey, we discovered three points. The first was that the percentage of mothers feeling loneliness did tend to decrease after seeing the Instagram ads. There are several studies that support our SNS approach. Shaw et al. reported that Internet communications can reduce the feelings of loneliness among adolescents and the elderly [23, 24]. Mandai et al. [25] showed that loneliness was significantly associated with having a smaller family social network (*β*=-0.32, *p* = 0.032), having fewer friends (*β*=-0.49, *p* = 0.001), and having a smaller SNS network (*β*=-0.21, *p* = 0.018). It has also been shown that the lower the awareness of social support the higher was the feeling of loneliness [26]. It is thought that a mother’s lack of a social network or her inability to recognize her network support is related to her higher degree of loneliness. It is possible that our message, that there is a place at her local consultation desk to connect with help and information, was successfully transmitted via the Instagram ads, which helped reduce the feelings of loneliness of some of the mothers. It is also thought that this type of message will help her in constructing personal networks similar to the connections made by traditional networks of family and neighborhood friends. This approach is consistent with the results of a previous study that found that higher individual-level social capital is associated with lower self-reported depressive symptoms [10].

The second point we found was that the participants with financial insecurity anxiety were significantly more likely to have seen and remembered the Instagram ads. The reason for this finding in not clear. And there is no previous research proposed about SNS and financial insecurity anxiety. Therefore it is needed the further study, but it suggested that when disseminating information using Instagram, a message that conveys how to better deal with financial instability might be useful and effective.

The third point was that about 90% our survey respondents used LINE, both those who normally use Instagram daily and those who do not. Instagram we used to deliver the ad in this study has been increasing the number of users most rapidly in Japan, but LINE has the largest number of users in Japan. [17] The range that Instagram advertisements can reach is thus more limited, and, going forward, it will be necessary to consider message delivery utilizing LINE.

There are several limitations within this study. First, the questionnaire survey targeting the group were different between before and after Instagram ads. In this study, the emphasis is on targeting with the mothers of same four-months children. If we targeted on same group of before and after the Instagram ads, the effect of the health check-up itself will be added and can’t interpret the effect of the ads intervention itself. We also thought that the randomized controlled trials were not suitable because the information spreads horizontally. Secondly, only 4.8% of mothers remember seeing Instagram ads and 8.6% remember Instagram ads in some way in this study. There is no previous study on the reach rate of Instagram ads, and it can be said that this study was able to clarify the actual situation, but unfortunately the effect of information transmission via Instagram this time may have been limited. There is also the possibility that Instagram ads have reduced the feeling of loneliness in some mothers, partly due to the horizontal transmission of information through friends. But it is hardly conclude that this Instagram ad has reduced loneliness of mothers with four months child. Furthermore, we may not be able to set a reliable and valid scale regarding loneliness and isolation. In this study, we focus on their feeling as an outcome. In next stage, after setting a reliable scale, we would like to evaluate the effectiveness of our “leaflet to reduce feelings of helplessness by making connections with local government offices” by reaching out to the target population.

Critically, the second half of the post-ad study, in March of 2020, coincided with the worldwide spread of COVID-19. It becomes difficult to interpret the impact of the ads because of not only the decrease in the number of examinees as lockdown was initiated, but also due to a wider range of factors that were influencing the mothers. We should also be cautious as to what extent the results of subjects undergoing regular health check-ups can be generalized. It is also necessary to consider messaging to those who have dropped out of regular health check-ups and to mothers who have children of different ages, as they also have feelings of loneliness. The continued spread of COVID-19 has severely restricted contact with family, friends, and healthcare, potentially leading to further isolation of mothers. In this study, we have shown the potential for social relationships via SNS to mitigate the sense of loneliness among mothers, and that it is necessary to establish new support methods using SNS for mothers of our next child-rearing generation.

## Conclusion

Our results indicate that there may be usefulness of SNS messaging for mothers raising small children in point of reducing their feeling of loneliness. Disseminating child-rearing information via Instagram may be effective, especially for people with financial instability. Furthermore, using LINE, which has many daily users, is an issue for future study. We strongly hope that this research will contribute to the promotion of maternal and child health.

## Supplementary Information

**Additional file 1.** The content of the questionnaire.

## Data Availability

All data generated and analysed during this study are included in this published article.

## Abbreviations

SNS: Social networking site
ads: Advertisements

## References

[1] Kenji Ogawa. Relationships and mental health counseling in the IT era. Kawashima shoten. 2018. p120-122. (in Japanese)

[2] Beutel ME, Klein EM, Brähler E (2017). Loneliness in the general population: prevalence, determinants and relations to mental health. BMC Psychiatry..

[3] Lee K, Vasileiou K, Barnett J (2019). Lonely within the mother’: An exploratory study of first-time mothers’ experiences of loneliness. J Health Psychol..

[4] Goto A, Quang Vinh N, Van Thi Tu, N, (2008). Maternal confidence in child rearing: comparing data from short-term prospective surveys among Japanese and Vietnamese mothers. Matern Child Health J..

[5] Cabinet Office. Section 2: Background of the causes of declining birthrates. https://www8.cao.go.jp/shoushi/shoushika/whitepaper/measures/w-2004/html_g/html/gg122000.html (Accessed on 1/10/2021)

[6] Ministry of Health, Labor and Welfare. The Overview of National Life Basic Survey. https://www.mhlw.go.jp/toukei/saikin/hw/k-tyosa/k-tyosa19/dl/02.pdf (Accessed on 3/3/2021)

[7] Ministry of Internal Affairs and Communications. Social life basic survey in H28. http://www.stat.go.jp/data/shakai/2016/pdf/youyaku3.pdf (Accessed on 3/3/2021)

[8] Cabinet Office. National Life White Paper on 2007: A rich national life that connection creates. Tokyo: Jiji Gahosha. 2007;1-118.

[9] Peplau LA and Perlman D (Eds.). Loneliness: A Sourcebook of Current Theory, Research and Therapy. (Wiley Series on Personality Processes). 1982;4–8.

[10] George K, Maria V, Vasiliki M, Vaggelis G, Anastassios EP, Panos B, Manolis K, Leda C, Antonis K (2013). Social capital in pregnancy and postpartum depressive symptoms: a prospective mother-child cohort study (the Rhea study). Intern J Nurs Stud.

[11] Earls MF, Yogman MW, Mattson G, Rafferty J (2019). Committee on Psychosocial Aspects 0f Child and Family Health. Incorporating recognition and management of perinatal depression into pediatric practice. Pediatrics.

[12] Smith MV, Lincoln AK (2011). Integrating social epidemiology into public health research and practice for maternal depression. Am J Public Health.

[13] Choi M, Mesa-Frias M, Nuesch E, Hargreaves J, Prieto-Merino D, Bowling A, Snith GD, Ebrahim S, Dale C, Casas JP (2014). Social capital, mortality, cardiovascular events and cancer: a systematic review of prospective study. Intern J Epidemiol.

[14] Boyd DM, Ellison N (2017). Social network sites: Definition, history, and scholarship. J Computer-Mediated Communication.

[15] Facebook. https://about.fb.com/ja/news/2021/04/2021-first-quarter-results/ (Accessed on 11/3/2021)

[16] Facebook. https://about.fb.com/ja/news/2019/06/japan_maaupdate-2/ (Accessed on 11/3/2021)

[17] Ministry of Internal Affairs and Communications, Institute for Information and Communications Policy. Survey report on information and communication media usage time and information behavior in 2018. https://www.soumu.go.jp/main_content/000644166.pdf (Accessed on 11/3/2021)

[18] Nolan S, Hendricks J, Ferguson S, Towell A (2017). Social networking site (SNS) use by adolescent mothers: Can social support and social capital be enhanced by online social networks? - A structured review of the literature. Midwifery.

[19] Mathiesen KS, Tambs K, Dalgard OS (1999). The influence of social class, strain and social support on symptoms of anxiety and depression in mothers of toddlers. Soc Psychiatry Psychiatr Epidemiol.

[20] Sperlich S, Arnhold-Kerri S, Geyer S (2011). What accounts for depressive symptoms among mothers?: The impact of socioeconomic status, family structure and psychosocial stress. Int J Public Health.

[21] Arimoto A, Tadaka E (2021). Individual, family, and community factors related to loneliness in mothers raising children less than 3 years of age: a cross-sectional study. BMC Womens Health..

[22] Qualter P, Munn P (2002). The separateness of social and emotional loneliness in childhood. Journal of Child Psychology and Psychiatry.

[23] Shaw LH, Gant LM (2002). In defense of the Internet: the relationship between Internet communication and depression, loneliness, self-esteem, and perceived social support. Cyberpsychol Behav.

[24] Chen Y-RR, Schulz PJ (2016). The effect of information communication technology interventions on reducing social isolation in the elderly: A systematic review. J Med Internet Res.

[25] Mandai M, Kaso M, Takahashi Y, Nakayama T (2018). Loneliness among mothers raising children under the age of 3 years and predictors with special reference to the use of SNS: a community-based cross-sectional study. BMC Women’s Health.

[26] Cacioppo JT, Fowler JH, Christakis NA (2009). Alone in the crowd: the structure and spread of loneliness in a large social network. J Pers Soc Psychol.

