# Supplementary material for: Development of information dissemination methods that contribute to improving maternal and child healthcare using social networking sites: a community-based cross-sectional study in Japan
Source: BMC Public Health. 2022 Mar 11;22:480. doi: 10.1186/s12889-022-12877-8 (PMC8913235; doi:10.1186/s12889-022-12877-8)
Supplement: Supplementary file 1 — Additional file 1. The content of the questionnaire. [file 12889_2022_12877_MOESM1_ESM.docx]

**Supplementary 1.** The content of the questionnaire.

| Please answer about yourself and your family. | |
| --- | --- |
| 1. | What is your current age? |
| 2. | Is child who have this checkup your oldest child?  - First-born, 2nd-born or later |
| 3. | Do you have any financial anxiety?  - Yes, no |
| 4. | Have you ever experienced a mental health problem?  - Yes, no |
| Please answer about your childcare situation for the child who had a checkup this time. | |
| 1. | How do you feel about your current child-rearing environment?  - I am satisfied, fairly satisfied, can't say either, not very satisfied, not satisfied |
| 2 | Is there anyone around you whom you can easily consult about child-rearing?  - Yes, no |
| 3 | Do you sometimes feel unconfident about raising your child?  - Often, rarely, can't say either way, not much, not at all |
| 4 | Have you ever felt lonely while taking care of children?  - Often, rarely, can't say either way, not much, not at all |
| 5 | Do you know the consultation desk in Takatsuki Health center?  - Already consulted, know but never consulted, not knowing |
| 6* | Do you know the message and information on the child-rearing consultation desk in Takatsuki City that have been posted in the advertisement section of Instagram from around December 2019??  - Seen, not seen but heard, not knowing |
| Please answer how you collect information in your daily life. | |
| 1 | What kind of SNS do you use in daily life?  - Facebook, LINE, Instagram, twitter, others, don't use |
| Please answer about the self-efficacy about your health. | |
| 1. | When you do something, you are confident.  - Definitely think so, somewhat think so, can't say either, not very think so, not think so |
| 2. | I feel anxious more easily.  - Definitely think so, somewhat think so, can't say either, not very think so, not think so |
| 3. | I often feel gloomy when I remember my past mistakes and unpleasant experiences.  - Yes, somewhat yes, can't say either, not very, no |
| Please answer about the self-efficacy of your child's health. | |
| 1. | When faced with a child's health problems, it is difficult to find an effective solution.  - Definitely feel so, somewhat feel so, can't say either, not very feel so, not fell so |
| 2. | Even if you plan your child's health, it usually doesn't work as planned.  - Definitely feel so, somewhat feel so, can't say either, not very feel so, not fell so |
| 3. | I feel that something good for the health of the children is being done.  - Definitely feel so, somewhat feel so, can't say either, not very feel so, not fell so |

- The Question included only in the questionnaire after posting the Instagram ads.
